# The Impact of a School-Based Water, Sanitation and Hygiene Intervention on Knowledge, Practices, and Diarrhoea Rates in the Philippines

**DOI:** 10.3390/ijerph16214056

**Published:** 2019-10-23

**Authors:** Hassan Vally, Celia McMichael, Claire Doherty, Xia Li, Gilbert Guevarra, Paola Tobias

**Affiliations:** 1 Department of Public Health, La Trobe University, Melbourne 3086, Australia; Claire.DOHERTY@svha.org.au; 2 School of Geography, The University of Melbourne, Melbourne 3053, Australia; celia.mcmichael@unimelb.edu.au; 3 Department of Mathematics and Statistics, La Trobe University, Melbourne 3086, Australia; X.Li2@latrobe.edu.au; 4 Australian Red Cross, Manila 1515, Philippines; gguevarra@redcross.org.au; 5 Philippine Red Cross, Manila 1515, Philippines; andreapaola.tobias@redcross.org.ph

**Keywords:** water, sanitation, hygiene, diarrhoea, school, Philippines

## Abstract

A school-based water, sanitation, and hygiene (WASH) intervention in the Philippines was evaluated. Students and households from four schools that received the WASH intervention (intervention schools) were compared with four schools that had not (comparison schools). Knowledge of critical handwashing times was high across all schools, but higher in intervention schools. Students reported higher rates of handwashing after toilet use (92% vs. 87%; RR = 1.06; *p* = 0.003) and handwashing with soap (83% vs. 60%; RR = 1.4; *p* < 0.001) in intervention versus comparison schools. In intervention schools, 89% of students were directly observed to handwash after toilet use versus 31% in comparison schools (RR = 2.84; *p* < 0.0001). Observed differences in handwashing with soap after toilet use were particularly marked (65% vs. 10%; RR = 6.5; *p* < 0.0001). Reported use of school toilets to defecate (as opposed to use of toilet elsewhere or open defecation) was higher among intervention versus comparison schools (90% vs. 63%; RR = 1.4; *p* < 0.001). Multilevel modelling indicated that students from intervention schools reported a 10-fold reduction in odds (*p* < 0.001) of school absence due to diarrhoea. In addition to school-based findings, self-reported handwashing at critical times was found to be higher among household members of students from intervention schools. This school-based WASH program appeared to increase knowledge and hygiene behaviours of school students, reduce absences due to diarrhoea, and increase handwashing at critical times among household members.

## 1. Introduction

In 2016, diarrhoea was the eighth leading cause of mortality among all ages causing approximately 1.66 million deaths, and a common cause of death among children aged under five years (approximately 446,000 deaths) [1]. Unsafe water and unsafe sanitation were leading risk factors for diarrhoea [1]. Although the majority of childhood deaths from diarrhoeal diseases are among children aged less than five years of age, there is a significant burden of diarrhoeal disease morbidity and mortality among school-aged children, particularly in low-income countries [2,3,4]. 

The WHO/UNICEF [5] state that a school with adequate water, sanitation, and hygiene (WASH) has a reliable, sufficient, and clean water supply; a sufficient number of toilets that are private, safe, clean, and gender segregated; handwashing facilities with water and soap; and hygiene education in the school curriculum. Facilities should cater to all, including small children, girls of menstruation age, and children with disabilities. Yet schools in many developing countries lack WASH services, with associated potential detrimental effects on health and school attendance. In 2016, only 57% of schools in the least-developed countries had adequate drinking water facilities and 53% had adequate sanitation [6]. 

Globally, WASH in Schools interventions aim to reduce the incidence of diarrhoea and other hygiene-related diseases; improve hygiene behaviours; improve school enrolment, school performance, and attendance; and influence the hygiene practices of parents and siblings via school students acting as agents of change [7]. 

There is evidence that water and sanitation infrastructure in schools are important foundations for reduced risk of WASH-related diseases, including diarrhoeal disease, respiratory illness, and soil-transmitted helminths, particularly when coupled with supporting hygiene promotion [2,8,9]. Hygiene promotion has been found to reduce WASH-related illness among school children [10]. Improved hygiene behaviours have been achieved through interventions that connect latrines to handwashing stations via pathways with brightly painted footprints, consistently provide soap and latrine cleaning materials, and deliver hygiene education to teachers and students [9,11,12]. There is some evidence that WASH interventions in schools reduce student absence by providing improved services (including for girls who are menstruating) and by reducing illness transmission [2,13,14,15,16,17]. Furthermore, there is evidence that WASH in Schools interventions reduce community disease burden and improve hygiene behaviours among students’ parents and younger siblings who are not attending school [14,18,19]. These studies caution that it is difficult to achieve behaviour changes among communities and family members due to broader conditions that limit improved behaviours (e.g., water scarcity).

Only a limited number of published studies have documented educational or health effects associated with provision or absence of water and/or sanitation and/or hygiene promotion in schools in low- and middle-income countries, with studies reporting findings from Kenya, Bangladesh, Cambodia, Ethiopia, India, Indonesia, Mali, Niger, Nepal, South Africa, Tanzania, and Turkey [3,7]. This paper focuses on the Philippines, a lower-middle income country. The Philippines is situated in Southeast Asia and consists of three main geographical regions: Luzon, the Visayas, and Mindanao. The Philippines has met or made ‘good progress toward’ its Millennium/Sustainable Development Goal targets for water supply and sanitation. As of 2015, 41% of the population had access to improved drinking water (MDG target met), and 39% of the 2015 population had access to improved sanitation. Yet in 2015, with a population of approximately 101 million people, 26% of the population still had no access to improved sanitation facilities, while 8% had no access to improved water sources [20]. In 2015 it was estimated that diseases related to poor water, sanitation, and hygiene (WASH) accounted for 15,000 deaths each year in the Philippines [21]. Consequently, WASH in Schools is an emerging priority in the Philippines.

Although this was a relatively small study with some methodological limitations, we believe this evaluation contributes to evidence of the effectiveness of WASH interventions in schools. The primary objective of the study was to evaluate the impact of a comprehensive school-based WASH intervention in elementary schools in La Union and Ilocos Sur provinces, Philippines. 

## 2. Methods

### 2.1. WASH in Schools Intervention

Philippine Red Cross (PRC) implements school-based WASH initiatives in elementary schools. Implementation of the PRC Expanded School-Based Water Sanitation and Hygiene Promotion project began in April 2013 in four provinces of Luzon. The project used the Children Hygiene and Sanitation Transformation (CHAST) methodology, which uses exercises and educational games to teach children about the links between personal hygiene and health. It is based on the premise that hygiene practices are acquired and readily shaped during childhood [22]. Schools were selected as beneficiaries of the intervention if they met the following criteria: (1) low access to water and sanitation facilities, (2) high incidence of water and sanitation related disease, and (3) no capacity to improve the situation. 

Water and sanitation infrastructure was built and restored in schools, including water storage systems, latrines, handwashing facilities, and water points. The capacity of schools to maintain the latrines and water points was developed via training and identification of school representatives to oversee maintenance. Consistent with CHAST methodology, students were equipped with knowledge on hygiene and prevention of water-related diseases. They were engaged in activities to improve their knowledge, skills, and behaviours in relation to health and hygiene. Both class-based and peer-to-peer hygiene promotion were a part of this intervention. Interactive sessions, peer-to-peer sessions, educational games, and arts and crafts activities were delivered to discuss, promote, and reinforce improved hygiene behaviours. Red Cross volunteers also distributed hygiene kits to students, which consisted of a toothpaste and toothbrush, hand soap, towel, nail clippers, comb, and cotton buds. Information, Education, and Communication (IEC) materials, (posters and flipcharts) were developed and distributed to support hygiene promotion and reinforce student learning. Each school formed a Water and Sanitation Committee, including the Principal, one health teacher, one Parent–Teacher Association (PTA) representative, and two focal students, to lead and oversee all WASH-related activities within schools. The Committees participated in two-day training to develop skills in conducting class-based and peer-to-peer hygiene promotion. 

### 2.2. Evaluation Design

This study was conducted between November 2016 and March 2017 in La Union and Ilocos Sur provinces in Luzon, Philippines. In this study, data were collected from eight public elementary schools: four schools that had recently completed the PRC WASH intervention (intervention schools) and four schools that had not received the WASH interventions (comparison schools) but were to be forthcoming beneficiaries. Schools were identified for inclusion in the evaluation if they were previous or forthcoming beneficiary schools, and were located in La Union or Ilocos Sur province. As such, schools were located within a specified geographic region (readily accessible from San Fernando, the capital of La Union province) and within a similar socio-political context. All schools included in this evaluation included both boys and girls from kindergarten to grade six (ages 5–12 years). All intervention schools had received the intervention approximately 12 months prior to this evaluation and thus the interventions were well established at the time of the evaluation. The evaluation of the impact of WASH interventions within the schools was based on a number of variables including the extent of water and sanitation facilities in schools, the assessment of hygiene knowledge and behavior, and self-reported rates of diarrhoea amongst students in schools. In addition, a survey of student’s households was completed to assess if WASH interventions in schools had any effect on household member handwashing behaviours and the rates of diarrhoea.

### 2.3. Data Collection

#### 2.3.1. Audit of Water and Sanitation Facilities

An audit of water and sanitation facilities (see UNICEF and WHO 2016) was conducted in each school which included, for example, the number/condition of toilets, water sources and availability, handwashing facilities, and waste disposal.

#### 2.3.2. Structured Observation of Student Handwashing

Structured observation of student handwashing following latrine use at each school was conducted. Teachers and principals were not told about the structured observation schedule in advance in order that they did not prompt students to wash their hands. Research staff positioned themselves unobtrusively to observe whether, and how, students cleaned their hands after they left the toilet. For each observation, field staff recorded the materials used to clean hands (e.g., water, soap, and/or sanitiser) and students’ gender. A minimum of 20 observations was recorded per school. 

#### 2.3.3. Student Survey of Hygiene Knowledge, Behaviours, and Diarrhoea Incidence

Student surveys were conducted with all children present at school on the day of data collection. In each classroom, students were provided with a paper-based survey (translated into Ilocano). Research assistants systematically explained each question to the younger students (taking care not to prompt responses). The survey collected data on self-reported hygiene knowledge and behaviours as well as the presence of diarrhoea and diarrhoea-related school absence in previous seven days. The WHO case definition of diarrhoea was used to define diarrhoea (three or more loose/watery stools in 24 h).

#### 2.3.4. Household Survey of Knowledge and Diarrhoea Incidence

Surveys were conducted in households of students attending schools. All students present on the day of the student survey were provided with a paper-based survey (translated into Ilocano) to give to an adult family member. To encourage completion and return, the survey was short (one page) and the first 60 students at each school who returned their survey were provided with a toothbrush and toothpaste. The survey collected household demographic information, information about household water and sanitation facilities, information about hygiene behaviours among household members, and cases of diarrhoea in the previous seven days.

### 2.4. Data Management

Data from student and household surveys were collected using paper-based methods and then entered into Magpi (home.magpi.com). Data were exported to Excel and cleaned.

### 2.5. Statistical Methods

Descriptive analyses of the audit of water and sanitation facilities, the observation of handwashing, and student and household survey were analysed in Excel. *T*-tests and chi-squared tests were used where appropriate to look for associations. A 5% level of significance was used for all statistical tests.

As the primary outcome in our study was diarrhoeal illness, multilevel models with random intercept for the student survey data were explored to describe the relationship between diarrhoeal illness and the intervention, adjusting for possible confounders. These models were performed in RStudio (Version 1.1.456, Boston, MA, USA) using the package lme4. Specific outcomes in these analyses were (1) presence of diarrhoea and (2) absence from school due to diarrhoea in the past 7 days.

Exploration of the data revealed that models were more stable when data were restricted to children ≥6 years of age. The original dataset had 1286 students. After deleting 13 students whose age was younger or equal to five years old, 1273 students remained. In the modelling stage, because of missing values, the numbers in the final models (with self-reported diarrhoea or absence from school with diarrhoea as the outcome) were 1259 and 1258 students.

Age, sex, and the presence of toilets in the household were forced into the model as these were obvious confounders. Multilevel modelling was used to take into account the clustering of schools. Sex was explored as a possible effect modifier, but this was not found to be the case. A model was explored in which one comparison school that appeared to be an outlier in terms of its poor conditions was excluded. However, removing this school from the analyses did not change the results, and so this analysis is not reported. Since hygiene knowledge and behaviours were secondary outcomes in this study, chi-squared tests were used to compare intervention schools with comparison schools.

### 2.6. Ethics

Research methods were approved by the La Trobe Human Ethics Committee and the Australian Red Cross (HEC16-098). Informed consent was sought from each school Principal and a member of the Parent–Teacher Association from each school. This process for student consent is standard procedure for the evaluation of WASH in schools, and is used by the Joint Monitoring Program (UNICEF/WHO), UNICEF, and the International Federation of Red Cross/Red Crescent. A plain language information sheet was reviewed verbally with all student participants prior to the conduct of the survey to clarify that responses were anonymous and to explain the purpose, themes, and processes of data collection. There is debate as to whether children can legally give informed consent, yet they can to assent to, or dissent from, participation [23]. Their completion of the survey, without any form of coercion, was an indication of their assent. Students were invited to take home a household-survey to be completed by a carer/parent. Written information about the survey was provided in plain language, making clear that participation was voluntary. Consent to participate in this survey was implied with the return of the survey.

## 3. Results

### 3.1. School Characteristics

All schools were public elementary schools that operated five days a week (Monday–Friday) and that included boys and girls from kindergarten to grade six (ages 5–12 years). The intervention schools were smaller than the comparison schools, with 692 students enrolled in the four intervention schools (average school size 173) and 1309 students enrolled in the four comparison schools (average school size 327). Consistent with their smaller size, intervention schools had considerably higher teacher to student ratios than comparison schools (5.7 teachers per 100 students vs. 3.7). The gender balances were comparable between the intervention and comparison schools, with 308 girls attending intervention schools (44.6%) and 650 girls attending comparison schools (49.6%).

### 3.2. Audit of Water and Sanitation Facilities

The results of the audit of water and sanitation facilities are summarised in Table 1. Intervention schools had a higher average number of functional taps per 100 students (6.6 vs. 1.7) and functional toilets per 100 students (8.1 vs. 2.2). All schools had a water source on site. All intervention schools had running water from faucets (via piped water or water pumped from a well). In contrast, two comparison schools relied on water from wells that were liable to run dry, one had water pumped from a well, and another accessed piped water. While all schools reported that water was usually available 5–7 days a week, every comparison schools reported that water was not sufficient. Additionally, one intervention school reported that it had a broken pump at the time of audit. All schools sold bottled water in their canteens. Soap was found to be available more often in intervention schools.

### 3.3. Direct Observation of Student Handwashing

**Figure 1:**
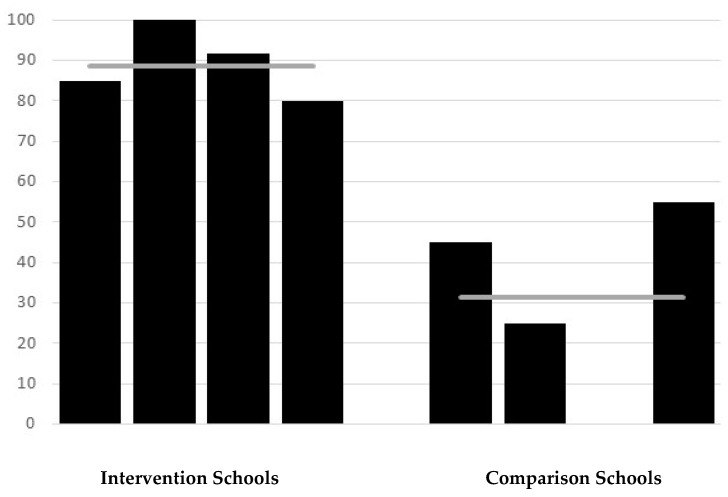
Direct observation of handwashing: percentage of children who washed hands after going to the toilet in intervention and comparison schools. Horizontal line represents the average for schools.

**Figure 2:**
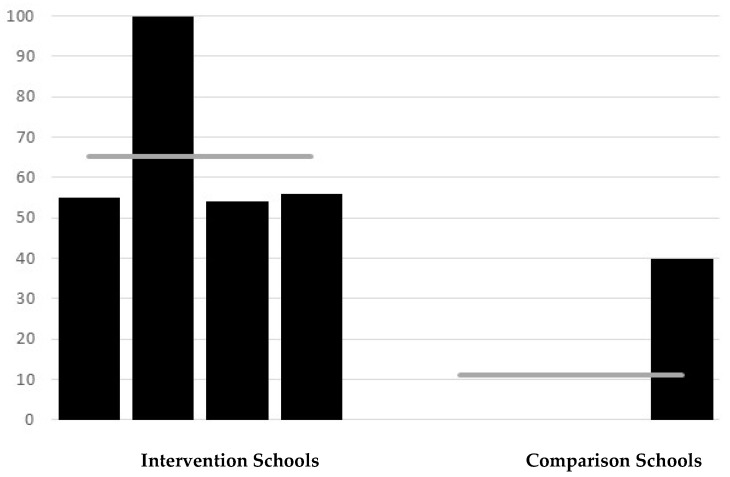
Direct observation of handwashing: percentage of children who washed hands with soap after going to the toilet in intervention and comparison schools. Horizontal line represents the average for schools.

A total of 169 observations of student handwashing were recorded (89 at intervention schools, 80 at comparison schools). Substantial differences between the intervention and comparison schools were observed in handwashing after using the toilet. On average, 89% of students from intervention schools were observed to wash hands after going to the toilet vs. 31% in comparison schools (RR = 2.84; *p* < 0.0001) (Figure 1). Handwashing rates were consistently high across intervention schools (range 80–100%) but varied amongst the comparison schools. In one comparison school, no child was observed to wash their hands after using the toilet, as the well was set apart from the toilet block and the water level was low with no pump, whereas 55% of children in another comparison school were observed to wash their hands after using the toilet, and here there was a functional tap near to the toilets. There were significant differences seen in direct observation of handwashing with soap, as to be expected given that three of the four comparison schools reported that soap was never available. On average, 65% of students from intervention schools were found to use soap when washing hands after going to the toilet compared with 10% from comparison schools (RR = 6.5; *p* < 0.0001) (Figure 2). In three of the intervention schools, about half of the students washed their hands with soap, with all students at the other intervention school using soap. Amongst the comparison schools, three schools were observed to have no students who used soap (soap was not available), and in one 40% of students were observed to wash hands with soap.

### 3.4. Student Survey of Hygiene Knowledge, Behaviours, and Diarrhoea Incidence

The size of schools, the number of responses received and the estimated response rates (based on students enrolled in the school) for the student survey are summarised in Table 2. The response rate for intervention schools was on average higher than for comparison schools, with 516 of 692 (74.6%) students from intervention schools completing the survey compared with 770 of 1309 (58.8%) students from comparison schools responding. The methodology of this survey (students were provided with questionnaires during class and these were collected on completion) ensured that the actual response rate of students at school on the day of the survey was close to 100% and so the estimated response rates reflect school attendance on the day of the survey. Notably, the two schools with the highest response rates (91.0% and 85.2%), and thus the highest attendance rates, were both intervention schools, while the two schools with the lowest response rates (42.0% and 65.9%) were both comparison schools.

Knowledge of critical times to wash hands was high in both intervention and comparison schools but consistently higher in intervention schools. Intriguingly, in both intervention and comparison schools relatively low rates of students correctly identified handwashing after toilet as a critical time (87.2% vs. 85.1%; RR = 1.02; *p* = 0.31), although this difference was not found to be statistically significant. In contrast, 98.6% of students from intervention schools correctly indicated that hands should be washed before eating, compared with 96.2% of students from comparison schools (RR = 1.02; *p* = 0.034). Similarly, 97.7% of students from intervention schools indicated correctly that hands should be washed prior to preparing and handling food compared with 90.2% of students from comparison schools (RR = 1.08; *p* < 0.001).

Self-reported handwashing with water (though not necessarily soap) after using the toilet was high for both intervention and comparison schools. However, more students from intervention schools self-reported that they ‘always’ wash their hands after using the toilet compared to students from comparison schools (91.7% vs. 86.6%; RR = 1.06; *p* = 0.004). (Table 3). As would be expected, due to reduced availability of soap in comparison schools, there was a greater difference between schools for self-reported handwashing with soap. Whilst 83.3% of students from intervention schools reported ‘always’ washing hands with soap, only 59.8% of students from comparison schools reported this (RR = 1.39; *p* < 0.001) (Table 4).

Students from intervention schools reported with much higher frequency that they used school toilets to defecate or urinate as compared to students from control schools (90.1% vs. 62.7%; RR = 1.44; *p* < 0.001) (Table 5). In contrast, students from comparison schools reported with much higher frequency that they defecated or urinated at another location outside the school toilets such as at home, anywhere outside the school, or in vacant lots.

Univariate analyses indicated students in intervention schools were approximately half as likely to report being ill with diarrhoea in the previous seven days compared to students from comparison schools (7.6% vs. 16.8%; RR = 0.45; *p* < 0.001). However, multilevel modelling, which takes into account the clustering of schools, did not confirm that the intervention was protective against self-reported diarrhoea (Table 6).

There were marked differences in self-reported absences from school with students from intervention schools having an approximately seven-fold reduced risk of being absent from school due to diarrhoea in the past 7 days based on univariate analyses (1.4% vs. 9.4%; RR = 0.15; *p* < 0.001). Multilevel analyses confirmed this association and indicated that the odds of being absent from school with diarrhoea in the past 7 days was 10-fold lower amongst students from the intervention schools versus those from comparison schools (OR = 0.10; CI 0.05–0.22; *p* < 0.001) (Table 6).

### 3.5. Household Survey of Hygiene Knowledge, Behaviours, and Diarrhoea Incidence

A total of 912 responses were received from households for this survey (402 responses from intervention households and 510 from comparison households). Given that 1286 students were in attendance at intervention and comparison schools on the days the household survey questionnaires were handed out, this represents an estimated response rate of 70.9%. This is very likely to be an underestimate of the response rate as many households had more than one child attending the school and so would have returned only one questionnaire. These estimated response rates varied significantly between the schools, with intervention schools providing response rates of 52.4%–93.1% (average 77.9%) and the comparison schools providing response rates of 29.3%–94.2% (average 66.2%).

All households from intervention school and comparison school catchment areas reported having access to water to wash hands. Similarly, all households from intervention school catchments (*n* = 401; 100%) and all but one household from the comparison schools’ catchments (*n* = 509; 99.8%) reported having access to soap for handwashing. There was a significant difference, however, between intervention and comparison catchment households in regard to the availability of toilets in the household. The frequency of households with toilets was much higher in intervention households (97.3%) than comparison households (81.0%). More detailed analyses of this discrepancy indicated that differences in the availability of toilets was attributable to the fact that one of the comparison schools was located in a far more disadvantaged socioeconomic context than the other schools in this evaluation. The number of households with a toilet in this disadvantaged school was 51.8% whereas the percentage of households with toilets in the other communities (including intervention and comparison communities) in this evaluation ranged between 95.2%–100%.

A significantly higher proportion of intervention households indicated that members ‘always’ washed their hands at critical times compared with comparison households (87.3% vs. 77.1%; RR = 1.13; *p* < 0.001) (Table 7). Twenty-three of the 402 surveyed intervention households (5.7%) reported that there was at least one case of diarrhoea in the past seven days among household members while 37 of 510 of the comparison households reported (7.2%) a case of diarrhoea. This difference was not found to be statistically significant.

## 4. Discussion

This evaluation of a WASH intervention in elementary schools in the Philippines comparing four schools that had received a WASH intervention with four that had not suggested that the school-based WASH intervention was effective across a number of measured outcomes, including the availability of adequate water and sanitation infrastructure, student hygiene knowledge and behaviours, and absence from school due to diarrhoea.

Water and sanitation infrastructure was considerably better in intervention schools, with higher numbers of functional taps (that supplied clean water on the day of survey) relative to student numbers and higher numbers of functional toilets. Although all schools were found to have a water supply that was usually functional, comparison schools all reported that water was not sufficient. Intervention schools reported that soap was sometimes available, whilst three of four comparison schools reported that soap was never available.

Also observed were self-reported differences in hygiene behaviours between intervention and comparison schools. However, given the infrastructure differences highlighted, it is impossible to tease out (through this study design) whether the educational interventions had effects on student hygiene behaviour change other than those related to the availability of infrastructure. This challenge has been noted in other cross-sectional studies that seek to evaluate multipronged interventions [8].

Rates of self-reported handwashing (with soap) after using the toilet were high amongst students of intervention schools, while lower, although not substantially lower, rates were reported in comparison schools. The observed differences in handwashing rates (20–25 observations per school) were found to be much greater than the self-reported rates with handwashing rates after using the toilet being observed to be much higher in intervention schools. While the use of soap when handwashing was low in all schools, very few students in comparison schools were observed to use soap for handwashing which is to be expected given the lack of soap in three of the four comparison schools. Whilst these findings necessarily reflect the variation in availability of water and soap for handwashing; they might also reflect improvements in hygiene education and knowledge.

One revealing finding from the student survey was where students reported that they defecated and urinated. Students from intervention schools were most likely to report that they used the school toilets, whereas students from comparison schools reported with higher frequency that they either went home, went to a location outside the school, or went in the vacant lot near the school. Again, this is a reflection at least in part of the considerable difference in WASH infrastructure between intervention and comparison schools.

This overall aim of these school-based WASH interventions is to reduce rates of diarrhoeal illness amongst students and, consequently, absence from school due to diarrhoea. Using multilevel modelling, students from intervention schools in this study were found to be have ten-fold reduced odds (OR = 0.1) of being absent from school with diarrhoea (*p* < 0.001). Although a reduced likelihood of self-reported diarrhoea was also found amongst intervention schools (OR = 0.29), this difference was not found to be significant.

Communicable illness is a leading cause of missed school days, and absenteeism is associated with reduced academic achievement [10]. Yet few studies have revealed a consistent relationship between diarrhoea (and other WASH-related illnesses) and student absence. For example, while some interventions that deliver handwashing promotion and point-of-use water treatment have reported reductions in days of student absence of between 21% and 54% [13,16], other studies have found no evidence of impact on attendance rates [9,24]. There are complex interactions between absenteeism and pupil health with the provision of improved WASH facilities potentially improving pupil attendance independent of the impacts on WASH-related disease. Hence WASH-related health effects may not be significant drivers of absenteeism in all contexts, and reduced faecal exposure among students via improved school WASH conditions may not be sufficient to reduce illness, particularly if household WASH conditions are poor [25]. It is interesting to note, however, that the estimated response rate for the student survey was a proxy for school attendance on the day of the survey, with a higher response rate in intervention schools relative to comparison schools. This considerable difference in school attendance may be attributed to a number of factors, but could be further evidence of reduced absences due to illness amongst students in intervention schools. Anecdotally we were told by school teachers and principals that students with prolonged or repeated absences due to illness were at significantly higher risk of not ever returning to school.

To explore children’s role and impact as WASH change agents in their households and wider communities, we conducted a household survey. This survey demonstrated a significantly higher rate of self-reported adherence to critical handwashing times amongst households of students attending intervention schools compared with comparison schools, providing some evidence to suggest that the benefits of school-based WASH interventions extended beyond school boundaries. Although we did not find a significant difference in the rates of diarrhoea in households for intervention schools compared to households for comparison schools, the power of this component of the study to show a difference in this outcome was somewhat limited.

### Limitations

There are several limitations to this study. Firstly, since this was a cross-sectional study comparing intervention and comparison schools, this precludes the demonstration of causal relationships. It is also possible that although differences in key outcome measures are likely to be largely due to aspects of the intervention, differences observed may have existed between the intervention and comparison schools before the interventions were impelmented. Although we feel this is an unlikely explanation for what was observed given the nature and the extent of the interventions, it cannot be discounted. It was also a limitation in this study that due to time and resource construants we were only able to enrol eight schools. Thus the small size of this study may have impacted in our ability to detect some effects, particularly the effect of the intervention on the presence of diarrhoea.

It was notable in this study that whilst there was some variation amongst comparison schools in the availability and quality of WASH infrastructure, one school (and community) was a clear outlier in terms of socioeconomic disadvantage. Given this, all statistically significant findings were tested for significance with this school both included and excluded from the analyses as a form of sensitivity analysis and only those findings where the exclusion of the disadvantaged did not change the overall findings were reported in this paper.

Another limitation is that the self-reported knowledge, behaviour, and health data could be subject to bias; indeed, it is evident from the differences between self-reported and observed handwashing that self-reporting (at least of hygiene behaviour) is unreliable. Despite efforts to conduct handwashing observation unobtrusively, there may have been differential reactivity by students during structured observation of handwashing at intervention and comparison schools. It is also important to note that the comprehensive nature of the WASH intervention means that it is impossible to assign any observed changes to particular components of the intervention, such as water and sanitation hardware or peer-led hygiene education. Further, the effectiveness of the intervention may have been associated with the typically unmeasured and unreported ‘exposure’ of intervention delivery and fidelity. At least one study has found that improved adherence to intervention design/delivery resulted in reduced prevalence of diarrhoea among pupils [26]. Intervention delivery and effectiveness depend on local participation and capacity staff, resulting in heterogeneity of implementation across different sites, which was unmeasured [8].

Although we feel that this study is a useful contribution to the literature on the effectiveness of WASH interventions, a longitudinal study with suffcient power to detect changes in outcomes such as absence due to diarrhoea would be valuable in determining the health and educational impacts of a comprehensive WASH in Schools intervention among primary school students.

## 5. Conclusions

The global effort to achieve sanitation and water for all by 2030 extends beyond the household to include institutional settings such as schools [6]. WASH in Schools seeks to improve student health, increase access to inclusive and effective education and learning, and contribute to health equity. The WHO and UNICEF established the Joint Monitoring Programme (JMP) for Water Supply, Sanitation, and Hygiene, which has developed global norms and indicators to benchmark and compare WASH progress, including in schools [6]. Nevertheless, there is no universal blueprint for effective WASH in Schools interventions and there remains a need to better understand the impacts of school-based WASH programmes, particularly in developing country contexts. Specific goals are to understand health and educational outcomes, to identify opportunities and challenges within program implementation, and assess intervention fidelity, to understand the extent to which students operate as WASH change agents in wider communities, and to consider the broader environmental and socio-political contexts that shape intervention outcomes [2,7,27].

The PRC has contributed to efforts to address and improve WASH in schools. The findings presented here suggest that students in intervention schools, compared to comparison schools, had improved WASH knowledge, hygiene behaviour-including handwashing after toilet use, and reduced absence due to diarrhoea. Students also appear to have played some role as change agents at the household level, with self-reported handwashing at critical times found to be significantly higher among household members from intervention schools. It needs to be acknowledged that this evaluation represents an assessment of a particular intervention, rather than of the potential for WASH in Schools to have an impact in any context. However, we feel that given the extent of the interventions that these findings are relevant to other low to middle income country settings. The results of this is evaluation suggest that school children are ready, reachable, and important targets for WASH in School interventions.

## Figures and Tables

**Table 1:** Audit of water and sanitation facilities in intervention and comparison schools.

Indicator	Intervention Schools	Comparison Schools
Mean functional taps per 100 students (range)	6.6 (4.5–11.5)	1.7 (0.3–3.4)
Number of non-functional taps (% of total taps)	2 (5%)	5 (19%)
Mean functional toilets per 100 students (range)	8.1 (5.4–14. 8)	2.2 (1.4–3.4)
Number of broken toilets (% of total)	1 (2%)	14 (35%)
Reported soap availability: number of schools (%)	Always: 0 (0%)	Always: 0 (0%)
	Sometimes: 4 (100%)	Sometimes: 1 (25%)
	Never: 0 (0%)	Never: 3 (75%)
Days that water is (usually) functional per week	5–7 days (100%)	5–7 days (100%)
When functional, water is insufficient: number of schools (%)	1 (25%)	4 (100%)

**Table 2:** Response rates for survey of students in intervention and comparison schools.

School	School Identifier	School Size (Girls, Boys)	Responses Received	Estimated Response Rate
Intervention Schools	I1	61 (24, 37)	52	85.2
	I2	223 (111, 112)	203	91.0
	I3	198 (83, 115)	137	69.2
	I4	210 (90, 120)	124	59.0
Total		692	516	74.6
Comparison Schools	C1	367 (179, 188)	242	65.9
	C2	282 (139, 143)	206	73.0
	C3	512 (258, 254)	215	42.0
	C4	148 (74, 74)	107	72.3
Total		1309	770	58.8

**Table 3:** Self-reported handwashing after toilet use.

Frequency	Intervention Households *n* (%)	Comparison Households *n* (%)	RR	95% CI	*p*-Value
Always	473 (91.7)	573 (86.6)	1.06	1.02–1.10	0.004
Mostly	35 (6.8)	75 (11.3)	0.60	0.41–0.88	0.005
Sometimes	8 (1.6)	12 (1.8)	0.86	0.35–2.08	0.46
Never	0 (0)	6 (0.9)	0.10	0.01–1.75	0.03 *

* Approximate RR and *p* value due to zero cell.

**Table 4:** Self-reported handwashing with soap after toilet use.

Frequency	Intervention Households *n* (%)	Comparison Households *n* (%)	RR	95% CI	*p*-Value
Always	430 (83.3)	396 (59.8)	1.39	1.30–1.50	<0.001
Mostly	74 (14.3)	194 (29.3)	0.49	0.38–0.62	<0.001
Sometimes	10 (1.9)	22 (3.3)	0.58	0.28–1.22	0.10
Never	3 (0.58)	53 (8.0)	0.07	0.02–0.23	<0.001

**Table 5:** Self-report of where students usually defecate or urinate during school hours.

Location	Intervention Households *n* (%)	Comparison Households *n* (%)	RR	95% CI	*p*-Value
In the school toilets	465 (90.1)	415 (62.7)	1.44	1.35–1.54	<0.001
At their own home	23 (4.5)	124 (18.7)	0.24	0.15–0.37	<0.001
Outside the school	25 (4.8)	106 (16.0)	0.20	0.3–0.46	<0.001
Vacant premises near school	4 (0.8)	87 (13.1)	0.06	0.02–1.16	<0.001

**Table 6:** Multilevel models showing the effect of the intervention on (1) diarrhoea in the past 7 days (n = 1259) and (2) absence from school in the past 7 days due to diarrhoea (n = 1258).

Predictors	Diarrhoea in the Past 7 Days			Absence from School in Past 7 Days due to Diarrhoea		
	Odds Ratios	CI	*p*	Odds Ratios	CI	*p*
Fixed effects						
(Intercept)	0.29	0.06–1.51	0.141	0.06	0.02–0.20	<0.001
Intervention	0.29	0.06–1.51	0.142	0.1	0.05–0.22	<0.001
Age of respondents	0.96	0.86–1.06	0.403	1.15	1.02–1.28	0.02
Sex	1.33	0.91–1.96	0.144	1.63	1.07–2.49	0.024
Toilets	0.45	0.20–1.02	0.056	0.53	0.28–1.03	0.062
Random Effects						
σ^2^	3.29			3.29		
τ_00_	1.08 _School_			0.00 _School_		
ICC	0.25					
N	8 _School_			8 _School_		
Observations	1259			1258		

**Table 7:** Frequency of those self-reporting that they ‘always’ wash hands at critical times in intervention and comparison households.

Critical Time	Intervention Households *n* (%)	Comparison Households *n* (%)	RR	95% CI	*p*-Value
After going to the toilet	351 (87.3)	393 (77.1)	1.13	1.07–1.20	<0.001
Before eating/preparing food	362 (90.1)	415 (81.4)	1.11	1.05–1.17	<0.001
After cleaning baby’s bottom	345 (85.8)	350 (68.6)	1.25	1.17–1.34	<0.001
Before feeding a child	357 (88.8)	370 (72.6)	1.22	1.15–1.31	<0.001
